# The effect of receptive music therapy on older adults with mild cognitive impairment and depression: a randomized controlled trial

**DOI:** 10.1038/s41598-023-49162-6

**Published:** 2023-12-13

**Authors:** Bing Xue, Xianmei Meng, Qiuxia Liu, Xianwu Luo

**Affiliations:** 1 School of Nursing, Wuhan University, 115 Donghu Road, Wuhan, 430071 Hubei China; 2 Peking Union Medical College Hospital, Chinese Academy of Medical Sciences, Beijing, China

**Keywords:** Ageing, Cognitive ageing

## Abstract

This study aims to evaluate the effects of a receptive music therapy intervention on cognitive functions and depressive symptoms in older adults with MCI. A randomized controlled trial was conducted in Wuhan, China. Eighty older adults, over 65, who had MCI and depression symptoms were randomly divided into the intervention and control groups. The intervention group received usual nursing care plus receptive music therapy intervention four times a week, for eight weeks; the control group received usual nursing care during the same period. The linear regression analysis was used to compare the difference between groups. There was a significant difference in the intervention group for the pre-intervention and post-intervention scores of cognitive function and depression. There was no significant difference in the control group before and after the study period and a significant between-group difference in both cognitive function and depression. In conclusion, receptive music therapy intervention significantly improved cognitive function and reduced depressive symptoms in older adults with MCI. It could be widely used in communities and nursing homes to improve the quality of life of older adults.

## Introduction

Mild cognitive impairment (MCI) represents a transitional stage between normal ageing and dementia, and the main symptoms are impairment of cognitive function, including memory loss and impairment of thinking and language functions^1–3^. MCI affects 10–15% of the population over 65 globally^4^, and China has one of the most rapidly ageing populations in the world. The number of older adults in China is 257 million, accounting for 17.9% of the total population^5^. A national research survey showed that the prevalence of MCI among older adults in China was 15.5%, indicating that roughly 38.77 million Chinese people have this disease^1^. MCI is a high-risk condition for progression to dementia^3^, which not only affects the daily life of older adults but exacerbates the burdens on society with tremendous impacts on health care and the economy^1^. China faces severe challenges in preventing, diagnosing, and treating dementia and related cognitive impairments^6^.

MCI is a risk factor for depression^7^; when cognitive impairment interferes with the ability to perform daily living, it can lead to the appearance of symptoms of depression^4^. The prevalence of depression in patients with MCI is high—roughly 32%^8^. Depression may accelerate the progression of MCI and lead to dementia^9,10^. Researchers found that patients with MCI concurrent with depression exhibit greater deficits in memory than non-depressed patients^11^, experience depression symptoms at approximately double the rate of healthy people^4^, and are more likely to have dementia^12^.

The use of music therapy for patients has grown in the past two decades^13^. Music therapy is associated with cognitive function and has been widely used to improve mental health^14,15^, memory, and executive function^13^. Music therapy is easily implemented and could be a potentially innovative strategy for improving cognitive function in older adults with MCI^14,16^. Receptive music therapy is a relatively simple way for participants to listen to music that they like and express their feelings^17^. A study demonstrated that receptive music therapy reduced behavioral and emotional problems in older adults with dementia, and was more effective than interactive music therapy^16^. And it was reported that music listening improved cognitive function and reduced depression symptoms^18^. However, one meta-analysis showed that music therapy had no significant effects on cognitive function^19^, while another meta-analysis found that only an active approach produced a positive effect^20^.

As a complementary therapy, music therapy is a powerful stimulus that can be used to regulate emotions and has provided beneficial effects for people with depression^21^. Music therapy administered by rehabilitation staff or nursing staff for patients with MCI can effectively improve their negative emotions^22,23^. Several studies have explored the effects of music therapy on older adults with MCI or older patients with depression^24,25^. However, the actual efficacy of music therapy in older adults is still controversial. For instance, one study found a significant improvement in depression in older adults^24^, while another showed no significant effect of music therapy on depressive disorder^26^. These contrasting results have led to uncertainty regarding the effectiveness of music therapy.

Previous studies have focused more on depression and dementia, and fewer studies have focused on this possible intervention for patients with MCI and depression^17,27^. One previous study showed that a music reminiscence activity had effects on both cognitive function and depression^28^. Additionally, due to different cultural backgrounds, there is still no clear evidence to prove whether music therapy is effective for patients with MCI and depression symptoms in China.

Studies have indicated that MCI is reversible; with early intervention, patients with MCI may return to a healthy condition^29^. Therefore, early intervention to improve cognitive function is very necessary. However, the guidelines of the American Academy of Neurology only state that regular exercise and cognitive interventions improve cognitive function^3^.

There is no clear evidence on the most effective format for music therapy. Populations of older adults with MCI and depression have not been fully studied and there are still no clear and effective intervention treatment plans. The purpose of this study was to evaluate the effects of this receptive music intervention on cognitive functions and depression symptoms in older Chinese adults with MCI and depression.

## Materials and methods

### Study design

An open-label randomized controlled trial was conducted in a nursing home in Wuhan China. This study was approved by the Wuhan University Institutional Review Board. The study was registered with the Chinese Clinical Trial Registry in 14/01/2022 (registration number: ChiCTR2200055614). All participants provided written informed consent. The data was collected by two nursing students who were trained to be familiar with the measurement procedures and the criteria for eligibility of the questionnaires. The receptive music therapy was conducted by a trained music therapist and an undergraduate nursing student who had studied nursing courses for older adults and music therapy. Since it was difficult to blind the participants in the intervention, only the outcome assessors were blinded to avoid the bias caused by subjective factors. This study was conducted following the CONSORT guidelines.

### Participants

All participants maintain a degree of independence in daily life, and are able to communicate, but lack the company of their children or other family members. All older adults in this nursing home have received neuroimaging tests. The sample size was calculated using G-power software version 3.1(Franz Faul, Germany). We hypothesized that the intervention had an effect size of 0.30. With a power of 80% and an α of 0.05, 37 participants for each condition of the study were required. Considering the percentage of loss in the sample, 40 participants for each intervention were determined. The inclusion criteria in this study were: (a) people older than 65 years; (b) presence of depression symptoms (15-item Geriatric Depression Scale score ≥ 8); and (c) presence of MCI (Montreal Cognitive Assessment score < 26). The exclusion criteria included: (a) history of cardiovascular disease, Parkinson's disease, or other severe physical or mental disease; (b) difficulty in moving and communicating; and (c) diagnosis of severe cognitive impairment such as dementia.

The 80 participants were randomly assigned using a computerized randomization scheme at a 1:1 ratio for the receptive music therapy intervention group (n = 40) and the control group (n = 40).

### Intervention program

The receptive music therapy intervention was carried out by a registered music therapist and an undergraduate nursing student who had studied nursing courses for older adults and music therapy. The music therapist was the primary interventionist, the undergraduate nursing student worked as an assistant. There were also another undergraduate nursing student and nursing home staff accompanied and monitored the participants.

Receptive music therapy usually consists of two types: relaxation and analytical, which are based on listening and encourage the expression and development of thought^30^. The music therapy program referred to previous studies^20,24,31^ to relieve depression improve cognitive function according to the purpose of this study. The intervention group came together to receive the musical intervention based on music listening, music discussion, and musical muscle progressive relaxation training 4 times a week for 8 weeks. A questionnaire was conducted before the intervention to select specific musical fragments for the participants based on their age, cultural background, and interests. The registered music therapist prepared the music after discussing the content with the staff in the nursing home, music therapists, and psychologists. Thus, the choice of music was tailored to individual needs and preferences. The music therapist played the music for the intervention group and encouraged them to discuss the music and share their stories. Music therapy sessions are conducted in the mid-morning in an open group format with the seniors sitting in a circle. During the course of the music therapy session, seniors are advised to close their eyes and visualize their happy memories. The intervention consisted of three steps. At the beginning of each session, each participant was warmly greeted, and the music therapist and the undergraduate nursing students would carefully determine the emotional and mental state of the participant. This would greatly influence the choice of music for this session. The first step was music listening, including music with a soft and cheerful melody, old-fashioned music, and songs. The music was played from a speaker. Each participant had an opportunity to choose individualized music that they like and the group listened to the music together for 20 min. The suitability of the playlists will be checked and documented during the music listening sessions and playlists will be continuously adapted over the intervention period as needed. The second step was music discussion, the registered music therapist invited the participants to give a verbal feedback on the experience and feelings. Participants were encouraged to share their stories related to the music. This part aimed to promote the communication abilities of the older adults and strengthen the relationship between them while reminding them of pleasurable memories such as marriage and family life. The third step was muscle relaxation. To the accompaniment of soothing music, the registered music therapist gradually guided the participants to relax the muscles throughout their bodies following the musical rhythm. The music was peaceful and recorded. Slow, minor tonalities, calm melodies, and no abrupt changes in the volume or rhythm. To achieve a relaxing effect, music such as Western tradition and light jazz were chosen as soothing music. The psychotherapist instructed the exercise.

Participants in the control group received the same treatment as the intervention group, minus the music therapy. This included routine daily care like health check-ups, regular outdoor activities, counselling, and health education. After the intervention period, the control group received the same music intervention as the intervention group.

### Outcomes

The primary outcome of this study was cognitive function. The secondary outcome was depression symptoms. The outcome measures were assessed at baseline and the end of the intervention. The Chinese-version Montreal Cognitive Assessment (MoCA) and the Chinese-version 15-item Geriatric Depression Scale (GDS-15) were used to assess cognitive function and depression symptoms, respectively, in the participants.

The MoCA has been widely used in the screening of MCI^32^. The assessment was translated into Chinese by Wang et al^33^. and the Cronbach’s α coefficient was 0.818^34^. There are seven testing dimensions in this tool including visuospatial/executive, naming, memory, attention, language, abstraction, and orientation. The total score obtainable is 30 points, and a score greater than or equal to 26 points is considered normal cognitive function.

Participants’ depression symptoms were measured by the GDS-15, which was a brief version of the Geriatric Depression Scale (GDS)^35^. There are 15 items on the scale and a total possible score of 15. An answer of “Yes” is generally scored as one point, but the first, fifth, seventh, and eleventh items use the reverse scoring method. A score higher than 8 points indicates the presence of depressive symptoms. The Chinese version of GDS-15’s specificity was 0.88 and the internal consistency Cronbach’s α coefficient was 0.82^36^.

The socio-demographic data included age, gender, education, current smoking status, current drinking habits, sleep status, frequency of learning or reading, frequency of exercise, presence of chronic disease and/or family history.

### Statistical analyses

All analyses were carried out using SPSS software version 20.0 (IBM Corp., Armonk, NY, USA). Statistical significance was defined as a two-sided *p* < 0.05. For the continuous information, we used mean and standard deviation. The sociodemographic characteristics between groups were compared by independent t-tests or chi-square tests to ensure comparability. We used a paired-sample t-test to compare the within-group difference from baseline to post-intervention. A linear regression analysis was used to evaluate differences in primary and secondary outcomes between the groups. We used forced entry, allowing all variables to be entered. We calculated the mean scores by intervention group and estimated difference using linear regression analysis. The pre-intervention scores of primary outcome and other sociodemographic variables (age, gender, education, smoking, drinking, sleep status, learning or reading, exercise, chronic disease, and family history) were included as covariates in the multiple regression analysis to control the confounding factors and account for the interindividual difference at baseline. The analysis included change in MoCA score as the primary dependent variable, as well as treatment group as the independent variable. The analysis of differences between groups for the secondary outcome was evaluated in the same manner with the addition of adjustment for baseline MoCA score. In each outcome, separate models were constructed. Interactions between the group and the outcome scores at baseline were explored to determine whether the intervention had an impact. All analyses were adjusted for baseline differences in outcomes of interest and the comparisons were presented based on the expected baseline adjusted mean differences with a 95% confidence interval between groups. An intention-to-treat (ITT) analysis was performed by using the imputation method, and all available data were used in analyses. Missing data were substituted by the mean scores.

### Ethics approval and consent to participate

This study was approved by the Institutional Review Board of School of Nursing, Wuhan University (WHU-2022-HL-004). All participants provided written informed consent and all methods were carried out in accordance with relevant guidelines and regulations.

## Results

**Figure 1:**
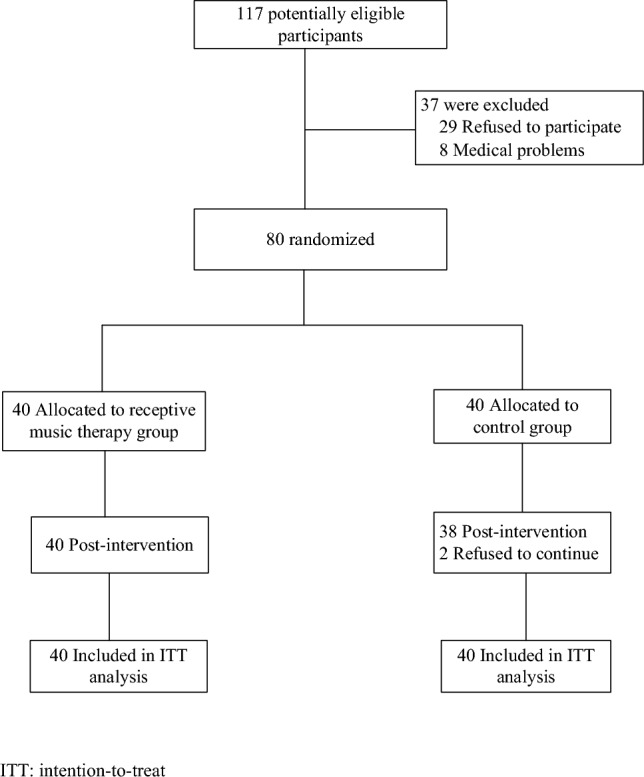
Flow chart of study participant selection and group allocation.

This study ran from February to June 2019. The initial survey was performed from February to March 2019 and the intervention was conducted from April to June 2019. A total of 117 questionnaires were distributed among the elderly in a nursing home in Wuhan and 80 elderly patients who met the inclusion criteria were included in the study (Fig. 1). All participants attended all sessions and none received any other treatment for cognitive function or depression during the intervention time. The multiple linear regression analysis showed a statistically significant effect in MoCA and GDS after controlling the sociodemographic variables such as age, gender, education, smoking, drinking, sleep status, learning or reading, exercise, chronic disease and family history.

The mean age was (75.43 ± 4.75) and (74.43 ± 4.47) years in the intervention and control groups, respectively. More than three-quarters of the participants were female and approximately half of them had only a primary school education with no degrees. More than half of the participants did not smoke or drink and most slept more than 6 h. Approximately half of the participants never learned or read after living in this nursing home while most of them consistently exercised. The participants in both groups had chronic diseases, but few of them had a family disease history. Before the intervention, the mean MoCA scores of the intervention group and control group were 18.03 ± 2.34 and 18.58 ± 2.34, respectively. The GDS-15 scores of the intervention group and control group were 9.15 ± 0.92 and 8.90 ± 1.43, respectively. There were no clinically relevant differences between the two groups at baseline (Table 1).

**Table 1:** Demographic characteristics of participants at baseline (n = 80).

Variables	Intervention group (n = 40)	Control group (n = 40)	*p*-value
Age(years) mean ± SD^†^	75.43 ± 4.75	74.43 ± 4.47	0.335
Gender			
Male	10 (25%)	8 (20%)	0.598
Female	30 (75%)	32 (80%)	
Education			
Primary school and no degree	21 (52.5%)	20 (50%)	0.549
High school or Polytechnic	14 (35%)	17 (42.5%)	
University degree	5 (12.5%)	3 (7.5%)	
Smoking	18 (45%)	14 (35%)	0.368
Drinking	12 (30%)	8 (20%)	0.308
Sleep status			
Less than 6 h	9 (22.5%)	7 (17.5%)	0.582
More than 6 h above	31 (77.5%)	33 (82.5%)	
Learning or reading			
Always	7 (17.5%)	7 (17.5%)	0.770
Little	12 (30%)	14 (35%)	
Never	21 (52.5%)	19 (47.5%)	
Exercise			
Always	32 (80%)	31 (77.5%)	0.788
Little	8 (20%)	9 (22.5%)	
Chronic disease	28 (70%)	24 (66.7%)	0.170
Family history	3 (7.5%)	2 (5%)	0.649
Score of MoCA (Mean ± SD) ^‡^	18.03 ± 2.34	18.58 ± 2.34	0.296
Score of GDS-15(Mean ± SD)^§^	9.15 ± 0.92	8.90 ± 1.43	0.355

^†^SD: standard deviation.

^‡^MoCA: Montreal Cognitive Assessment.

^§^ GDS-15: 15-item Geriatric Depression Scale.

SD: standard deviation.

Table 2 presents the primary and secondary results. Post-intervention, the mean MoCA scores of the intervention and control groups were 20.65 (SD = 2.41) and 18.55 (SD = 2.36), respectively. There was no significant difference between baseline and post-intervention in the control group (*p* > 0.05). The MoCA score in the intervention group was significantly higher than before the intervention (mean difference = 2.63, 95% CI = [2.09, 3.17], *p* < 0.05). Compared to the changes in the control group, there was a statistically significant difference between groups, with the intervention group’s MoCA scores notably higher than the control group’s (adjusted mean difference = 2.41, 95% CI = [1.50, 3.31], *p* < 0.001). The details of each MoCA item are shown in Appendix 1.

**Table 2:** Comparison and distribution between assessment time of the intervention group and control groups (n = 80).

Outcomes	Control group (M ± SD)	Intervention group (M ± SD)	MT group vs control group	*P* value
			Adjusted mean difference (95%CI)^§^	
MoCA				
Baseline	18.58 ± 2.34	18.03 ± 2.34	–	< 0.001
Week 8	18.55 ± 2.36	20.65 ± 2.41	2.41 (1.50 to 3.31)	
Mean difference(95%CI)^†^	− 0.03 (− 0.89 to 0.84)	2.63 (2.09 to 3.17)		
*P* value	0.954	< 0.001		
GDS-15^‡^				
Baseline	8.90 ± 1.43	9.15 ± 0.92	–	< 0.001
Week 8	8.88 ± 1.42	6.33 ± 1.86	− 2.54 (− 3.28 to − 1.79)	
Mean difference(95%CI)^†^	− 0.03 (− 0.75 to 0.70)	− 2.83 (− 3.43 to − 2.22)		
*P* value	0.945	< 0.001		

MoCA: Montreal Cognitive Assessment, GDS-15: 15-item Geriatric Depression Scale, M: mean; SD: standard deviation; CI: confidence interval.

^†^Pairwise comparison between assessment time of the intervention group and the control group from baseline to post-intervention.

^§^Intervention effects are quantified as differences between groups in the mean value for each scale, estimated using a multiple linear regression model. All regressions included socio-demographic data (age, gender, education, smoking, drinking, sleep status, learning or reading, exercise, chronic disease, family history) and baseline scores of MoCA and GDS-15.

For the GDS-15, the mean scores of the intervention and control groups were 6.33 (SD = 1.86) and 8.88 (SD = 1.42), respectively. There was no significant difference from baseline to post-intervention in the control group (*p* > 0.05). However, the intervention group’s GDS-15 scores post-intervention were significantly lower before the intervention (mean difference =  − 2.83, 95% CI = [− 3.43, − 2.22], *p* < 0.001). There was a statistically significant between-group difference for GDS-15 scores, with the intervention group lower than the control group (adjusted mean difference =  − 2.54, 95% CI = [− 3.28, − 1.79]). There was a between-group difference in the intervention and control groups in both MoCA and GDS-15 scores. The effects were transformed into effect sizes and were interpreted in accordance with a previous study (less than 0.2 was small effect, from 0.2 to 0.5 was medium effect, more than 0.8 was large effect^37^).The effects of receptive music intervention were clinically relevant, with effect sizes in the medium level (ranging from 0.36 for depressive symptoms to 0.40 for cognitive function). Effects on the two outcomes were smaller than medium.

## Discussion

The participants in the music intervention group improved significantly in both objectively scored cognitive function and depressive symptoms; however, no statistically significant changes were observed in the control group. This study provided evidence that receptive music therapy intervention had a positive effect on cognitive function and depression symptoms in older adults with MCI. This confirms that it can be a useful intervention for older adults with MCI and depression symptoms.

Our results indicated that the MoCA scores in the intervention group were significantly higher after receptive music therapy intervention and cognitive function was improved, but there was no significant change in the control group, providing preliminary evidence that receptive music therapy intervention effectively improved cognitive function. The result indicated that a music-based intervention was associated with improved cognitive function in older adults with mild to moderate cognitive impairment, which is in line with a previous study^38^. Several studies have demonstrated that listening to music can improve learning, memory, and cognitive function in older adults with MCI^22,39^. Music interventions may induce changes in the frontal and parietal cortex, which exerts beneficial effects on the memory of lyrics, and an individual’s digit span and working memory^20,40^. Studies have found that the use of familiar music in music intervention was able to stimulate participants’ autobiographical memory; enhance patients’ communication and learning abilities; and improve patients’ cognitive functions^41,42^.

Our results indicated a significant difference between pre-and post-intervention GDS-15 scores in the intervention group. Music therapy can effectively reduce depression and is an important intervention. A study found that music therapy intervention decreased depression levels in older adults^24^, which was similar to our findings. Music therapy can reduce α waves in the brain, allowing older adults to relax and improve their independence—thus enhancing their self-confidence^24^. Our study encouraged the participants to discuss and share their stories, since sharing stories related to music help improve mood and promote social interaction among older adults^43^. Previous studies reported that receptive music intervention for MCI treatment is mainly applied through the effects of the rhythm and soothing characteristics of music on the human body, relaxing the patient’s mind and finally affecting brain function^22,42^. Finn and Fancourt^44^ found that almost 40% of the biomarkers they tested changed due to music listening, and half of the clinical studies have proven the decompressive effect of music listening. Receptive music therapy was previously used in MCI treatment, which mainly affected the patient through the rhythm and soothing characteristics of music, enabling the patient’s whole body and mind to be in a state of relaxation and harmony, and ultimately affecting brain function^18^. It mainly stimulateed the emotions of older adults through the method of listening, which can awaken connection and integrate personality, as well as enhance cognition of self and differentiation between self and others^22^. Depression affects the expression of brain-derived neurotrophic factor (BDNF), leading to the atrophy of neurons and the hippocampus^45^. Hippocampal activities have been proven to be associated with learning, memory and spatial orientation, and hippocampal formation also has been shown in response to music-evoked joy^46^. Music enriches the social support environment, which is associated with increased BDNF levels^47^. A previous study found that the serum BDNF levels of patients receiving music therapy were higher than that of patients who did not receive music therapy, suggesting that music therapy can promote the formation of serum BDNF, helping to repair nerve function^48^. Listening to music is associated with changes in the frontal lobes and limbic areas, which are associated with the processing of visual information^46^; and BDNF is upregulated in both music listening and performance, which can improve cognitive function and mood.

The receptive music therapy intervention can be conducted in a variety of places (such as community centers, nursing homes, and participants’ homes), in groups, or individually^21^, be active or receptive, and this intervention is inexpensive and easy to implement. It is worth promoting in both nursing homes and communities. Research exploring early intervention strategies for MCI noted that this intervention was the safest intervention for MCI, although only moderate effects were suggested^22,48,49^. In this study, the implementation of music therapy intervention was feasible, the participants attended all meetings, high retention rates were achieved, and no adverse events were reported.

This study has several limitations. First, the study only assessed the effects after the intervention. The long-term effects of music therapy were not evaluated. Second, due to the lack of staff, we chose group intervention. And we encouraged the expression and development of thought, which were parts of the receptive music therapy. While this can help the elderly to communicate and share their stories, it was difficult determining whether the music alone was effective because communication of group meeting can be a confounding variable for depression. However, the control group also received social or group component such as the outdoor activities, counseling and health education intervention which may reduce the confounding effect. What’s more, the measurement of cognitive function was conducted using the MoCA, which is often used for screening. Future research could benefit from the incorporation of a neuropsychological assessment tool. In the future, follow-up studies should further explore the significance of this intervention, more types of interventions need to be developed, and more registered music therapists should be involved in the development of interventions.

In conclusion, this study explored the effects of a receptive music therapy intervention on older adults with MCI and depression in China. Our study provided promising results for improvement in cognitive function and depression symptoms. The receptive music therapy intervention was easy to intervene and interesting for participants, ensuring better compliance, and allowing all participants to complete the study. The 8-week receptive music therapy intervention had positive effects on older adults with MCI and depression. Thus, the receptive music therapy intervention is suitable for promotion in both communities and nursing homes. However, receptive music therapy intervention still needs to be verified in further research exploring how it could delay or reduce the occurrence of MCI and improve the quality of life in older adults.

## Data Availability

The data that support the findings of this study are available on request from the corresponding author.

## Appendix 1

See Table

**Table 3:** Mean differences for MoCA subscale scores from baseline to post intervention in the music therapy group.

MoCA subscales	Mean difference (95%CI)	*p* value
Visuospatial/executive function	− 0.15 (− 0.35, 0.05)	0.135
Naming	− 0.05 (− 0.12, 0.02)	0.16
Memory	− 0.90 (− 1.28, − 0.53)	< 0.001
Attention	− 0.63 (− 0.92, − 0.33)	< 0.01
Language	− 0.13 (− 0.29, 0.04)	0.133
Abstraction	− 0.45 (− 0.67, − 0.23)	< 0.001
Orientation	− 0.15 (− 0.32, 0.02)	0.083

^3^.
